# Editorial: Neuroendocrine regulations in fish: role of environmental and internal factors

**DOI:** 10.3389/fendo.2024.1470816

**Published:** 2024-08-15

**Authors:** Romain Fontaine, Paula Gabriela Vissio, Dianne M. Baker

**Affiliations:** ^1^ Department of Preclinical Science and Pathology, Faculty of Veterinary Medicine, Norwegian University of Life Sciences, Ås, Norway; ^2^ Facultad de Ciencias Exactas y Naturales, Departamento de Biodiversidad y Biología Experimental, Universidad de Buenos Aires, Buenos Aires, Argentina; ^3^ Instituto de Biodiversidad y Biología Experimental y Aplicada (IBBEA), CONICET, CONICET – Universidad de Buenos Aires, Buenos Aires, Argentina; ^4^ Department of Biological Sciences, University of Mary Washington, Fredericksburg, VA, United States

**Keywords:** fish, endocrinology, neuroendocrinology, reproduction, growth

## Introduction

It is well established that in vertebrates, including fish that represent more than half of the extant vertebrate species (1), the brain-pituitary axis plays a pivotal role in controlling growth, metabolism, homeostasis, reproduction, metamorphosis, and stress responses. Located below the hypothalamus, the pituitary is characterized by its high plasticity, which is essential for adapting hormone production to meet demands based on environmental conditions and the animal’s life cycle. Yet research is still revealing new components and mechanisms that participate in endocrine regulation and plasticity. For instance, cholecystokinin, long known for its role in digestion and appetite regulation, has only recently been characterized as also functioning as a new follicle-stimulating hormone (Fsh) releasing hormone in fish (2, 3). Additionally, recent studies show that different endocrine axes, once thought to be independent, interact within a complex network. This is exemplified by the thyrotropic and gonadotropic axes in regulating reproductive functions. For instance, it was just demonstrated that thyroid-stimulating hormone (Tsh) can stimulate the proliferation of gonadotrope cells in fish (4). Studies investigating when in the life cycle or under which environmental conditions this plasticity plays a role, the cellular and molecular mechanisms allowing it, and how these mechanisms are regulated have intensified in the last decade due to more powerful methodological tools.

On this basis, we conceived and coordinated the publication of a Research Topic on “Neuroendocrine Regulations in Fish: Role of Environmental and Internal Factors.” This collection presents five original research articles, covering the different mechanisms involved in the neuroendocrine regulation of growth and reproduction.

## Mechanisms for neuroendocrine regulation

The first paper, “Simultaneous Extraction and Detection of Peptides, Steroids, and Proteins in Small Tissue Samples,” by Lu et al., presents a methodological advancement for the simultaneous measurement of peptides, steroids, and proteins in small tissue samples. Utilizing high-resolution liquid chromatography tandem mass spectrometry, the authors demonstrate the sensitivity and efficacy of their novel method for measuring several signaling molecules in the brain, pituitary gland, and gonads of single zebrafish samples. This technique allows for a more integrated understanding of hormonal pathways and their interrelationships, which is crucial for advancing our knowledge of neuroendocrine regulation and its impact on fish physiology.

The second paper “Mutation of Brain Aromatase Disrupts Spawning Behavior and Reproductive Health in Female Zebrafish,” by Shaw et al., investigates the roles of the aromatases, steroidogenic enzymes critical for estrogen synthesis, in zebrafish reproduction. The study uses *Cyp19a1 -/-* mutant zebrafish lines to elucidate the importance of two aromatase paralogs, *cyp19a1a* (expressed in the gonads) and *cyp19a1b* (expressed in the brain and pituitary), in reproductive behavior, offspring survival and early development. Shaw et al. report that mutation of *cyp19a1b* increases the latency to oviposition and affects egg production and early progeny survival. This research underscores the essential role of the brain-expressed paralog in not only spawning behavior, but in other roles contributing to reproductive success in teleost fish.

The third paper, “Neurohypophysial and Paracrine Vasopressinergic Signaling Regulates Aquaporin Trafficking to Hydrate Marine Teleost Oocytes,” by Ferre et al., explores the hormonal regulation of oocyte hydration in marine teleosts, a key adaptation for reproductive success in ocean environments. Using the gilthead seabream as a model, the study focuses on the roles of arginine vasopressin (Avp) and oxytocin (Oxt) in this process, revealing that these nonapeptides circulate systemically but are also produced locally in the ovarian follicles during oocyte maturation. Functional characterization of Avp and Oxt receptor subtypes shows their involvement in activating signaling pathways that regulate aquaporin insertion in oocyte membranes. These findings provide evidence of another role and source of a classic hormone in marine teleost reproduction.

The fourth paper, “Cysteamine Improves Growth and the GH/IGF Axis in Gilthead Sea Bream (*Sparus aurata*): *In Vivo* and *In Vitro* Approaches,” by Sánchez-Moya et al., examines the effects of cysteamine (CSH) as a feed additive on growth performance and the growth hormone/insulin-like growth factor (GH/IGF) axis in gilthead sea bream. The study demonstrates that CSH enhances growth, with evidence that this growth may be mediated by modulating the GH and IGF signaling pathways in different tissues (liver, stomach, and white muscle), promoting an anabolic state. Thus, the authors provide evidence that CSH could be a promising dietary supplement for enhancing fish growth in aquaculture. These findings have significant implications for improving fish production and understanding the hormonal regulation of growth in teleosts.

The last paper, “Characterization of the Somatostatin System in Tilapia: Implications for Growth and Reproduction,” by Mizrahi et al., explores the roles of somatostatin (SST) in regulating growth hormone secretion and reproductive hormone levels in tilapia. The study identified three SST peptides and four isoforms of the somatostatin receptor (SSTR) in the tilapia genome. Using RNA sequencing and *in situ* hybridization, the authors reveal the expression of these peptides in the brain and the receptors in the pituitary cells, including in gonadotropes. Notably, the study shows that somatostatin receptors influence gonadotropin release *in vivo*, which has implications for managing growth and reproductive health in cultured fish populations. These findings provide a foundation for further research into the complex and interlinked neuroendocrine control of reproduction and growth in fish.

## Conclusion

This Research Topic offers an update on different aspects of neuroendocrine regulation in fish, highlighting novel paradigms evidenced in primary research manuscripts. By exploring the roles of specific hormones, receptors, and signaling pathways, the papers collectively provide valuable insights into the complex neuroendocrine mechanisms that regulate growth and reproduction in fish. We hope that this compendium will be useful to researchers interested in vertebrate neuroendocrinology and contribute to the development of more effective strategies for promoting growth and reproductive success in both wild and cultured fish populations.
